# Treatment of Chronic Prostatic Enlargement

**Published:** 1881-12

**Authors:** 


### Treatment of Chronic Prostatic Enlargement.

Mr. Thos. Smith, surgeon to Bartholomew's Hospital, in a recent lecture published in the London *Medical Times and Gazette*, gives the following advice on the above subject:—

*Treatment.*—Your assistance will rarely be sought in the early stages of this disease; but should you be consulted by an elderly patient suffering from undue frequency or difficulty in micturition, it will always be prudent to make a digital examination through the rectum, to ascertain the condition of the prostate. The examination is best made with the patient lying down on his back. Your finger-nail being filled with soap, and the finger well oiled or greased, it should be introduced very slowly, so as not to excite spasm of the sphincter.

Should you judge that the urinary difficulty is caused by prostatic enlargement, the occasional passage of a full-sized instrument will often relieve the inconvenience, and, if steadily persevered in at regular intervals, will generally secure the patient against all the more serious consequences of the disease.

In cases where the difficulty in micturition has gone on to produce an inability to empty the bladder completely, it is of primary importance that at least once in twenty-four hours the urine should be all drawn off; but in carrying out this plan it is necessary to exercise caution, lest by suddenly emptying a greatly distended bladder you should produce a complete paralysis of the organ, with a loss of the power of voluntary micturition, and cystitis.

As a general rule, if there be not more than one pint of retained urine in the bladder—that is, urine the patient is unable to pass for himself, it may be safely drawn off at once. But if there be more than this of residual urine (and there may be several pints), you should draw it off by installments, taking away a little more each day, until the bladder is completly emptied.

This complete evacuation of the bladder, when once accomplished, should be repeated each day, by means of an instrument, and for the purpose an india-rubber catheter, a bulbous-ended or Coude catheter, should, if possible, be used.

By these means, in an early stage of the disease, the patient will generally regain the power of normal micturition, or at all events, if this result be not attained, he will be secure from the worst consequences of the disease.

The treatment may be carried out by the patient himself if you will be at the pains to teach him how to pass an instrument—nowadays a comparatively simple process, owing to the great improvements in catheters; for you should know that since the introduction of the various forms of soft catheters now in use, the instrumental treatment of prostatic enlargement has lost more than half its terrors and much of its danger.

This large silver prostatic catheter which I now show you—at one time almost the only instrument used in these cases—is truly a formidable weapon with its long shaft and wide-sweeping curve. It was constructed to ride over the prostate, but in the hands even of experienced surgeons it frequently failed in the performance of its normal functions and rode under the gland, or through its substance. Used with a strong and steady hand it rarely failed to draw off water. As an instance of its powers in this respect, I may mention a case within my knowledge where a prostatic catheter in the hands of an energetic surgeon drew off some gallons of water, which, however, a post-mortem examination disclosed to have come from the peritoneal cavity.

I will suppose now that you are called upon to treat a patient with retention of urine dependent upon enlarged prostate. The difficulty will usually have come on at night time; the patient will, as a rule, be advanced in years; and the prostate can be felt in the rectum unduly prominent. In such a case let me advise you first to try a flexible red rubber catheter, of full size; it will often find its way round a corner, and through a urethra which would be impervious to a more rigid instrument. This failing, you should try and pass the same catheter with a stout wire stylet reaching two-thirds of the way down the instrument; this gives you more power to push the catheter onwards, and leaves the end flexible, to accommodate itself to the distorted urethra.

Next in order you may try the Coude catheter, which I show you: then, if necessary, the bulbous French instrument, a gum elastic, without and with the stylet; and lastly, others failing, a silver instrument.

Whatever instrument you use, let it be a full size; it will go in as easily as a smaller one, and is less likely to damage your patient. Keep the point of the instrument on the upper wall of the urethra; and, above all things, use no force.

After drawing off the water in a case of retention, the patient will, for a time at least, require the regular use of the catheter until he recover his power of voluntary micturition; and should there have been great difficulty in introducing the catheter, I should advise you to tie it in for the first twenty-four hours.

In the subsequent treatment of these cases of prostatic retention, in addition to other troubles, you will often have to contend against an increasing frequency in micturition. The frequent desire to pass water must be resisted as much as possible by the patient, or it will grow upon him. The bladder must be completely emptied, and, if need be, washed out, at regular intervals, and the patient exhorted not only to resist by a strong effort of the will the solicitations of his bladder, but to avoid all sights and association that are likely to suggest to him the necessity of micturition. With this object in view, you should counsel your patient to keep his catheter and chamber-utensil out of sight; as soon as possible to leave his bed-room during the day; and to occupy his mind by any pursuit which may draw his thoughts away from his urinary necessities.

